# Supplementary material for: DMRforPairs: identifying Differentially Methylated Regions between unique samples using array based methylation profiles
Source: BMC Bioinformatics. 2014 May 15;15:141. doi: 10.1186/1471-2105-15-141 (PMC4046028; doi:10.1186/1471-2105-15-141)
Supplement: Additional file 2 — DMRforPairs output for the comparison of A431-MCF7 and NA17018-NA17105. Please start from the HTML files in each folder. Available via the BMC Bioinformatics website. [file 1471-2105-15-141-S2.zip › 1394847754114233_MOESM2_ESM/A431_MCF7/figures/1183.pdf]

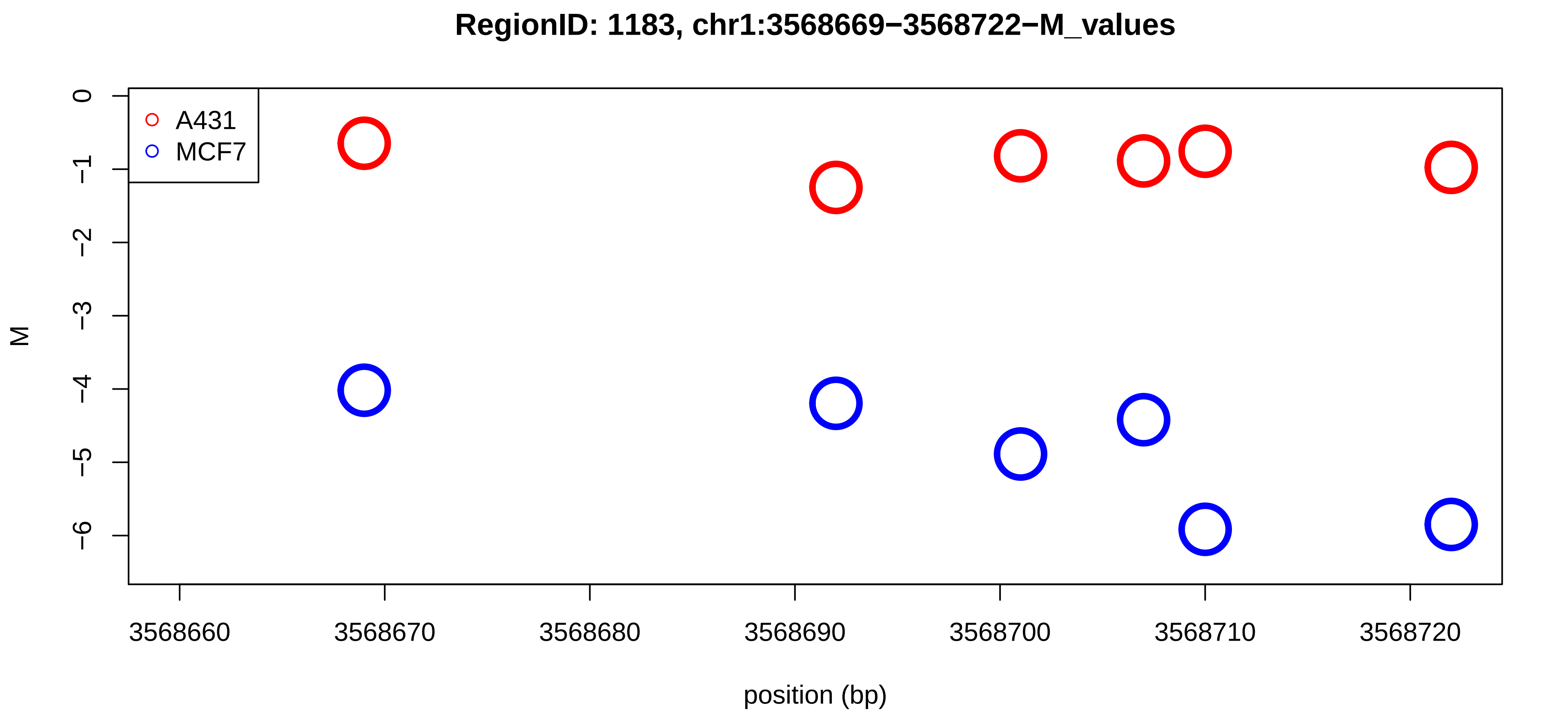
RegionID: 1183, chr1:3568669−3568722−M_values
A431
MCF7
M
0
−1
−2
−3
−4
−5
−6
3568660
3568670
3568680
3568690
3568700
3568710
3568720
position (bp)

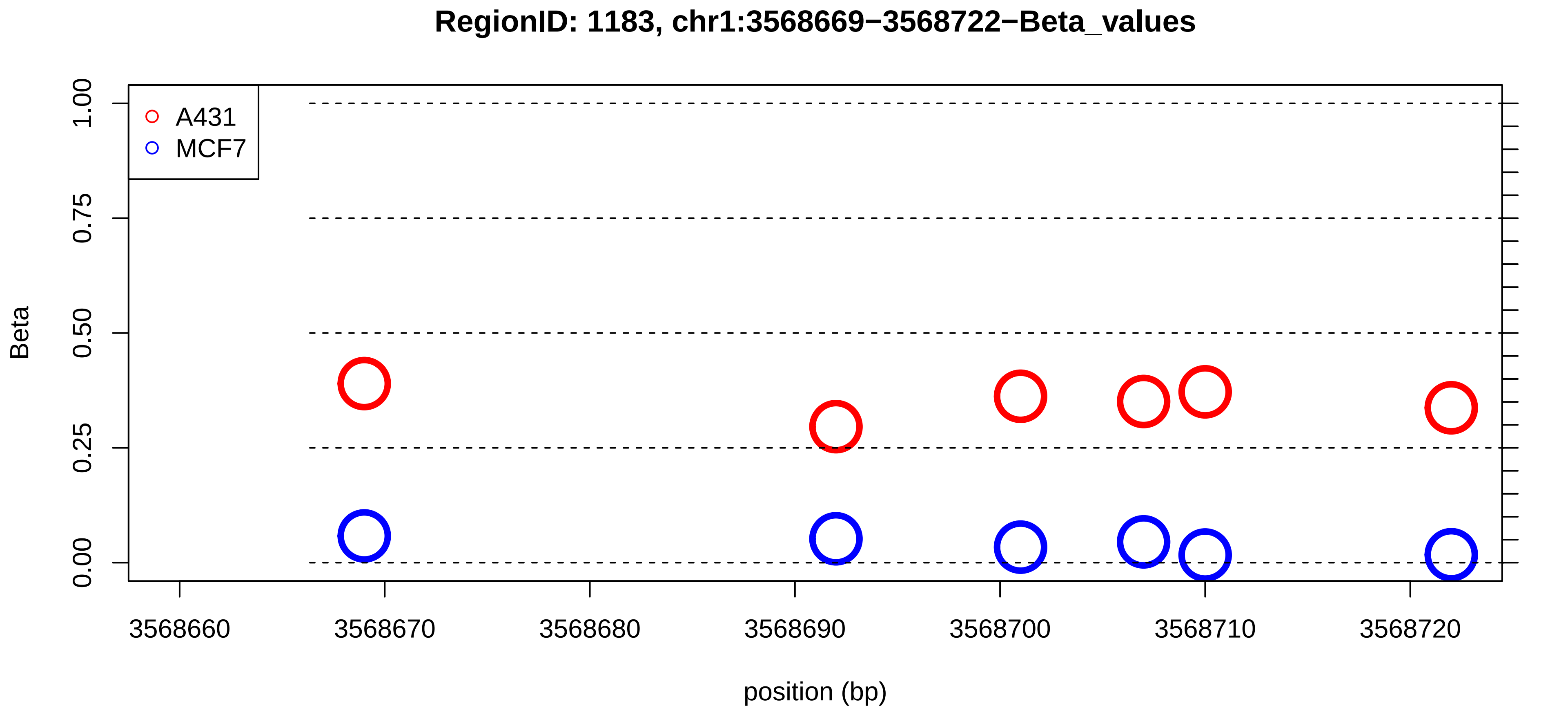
RegionID: 1183, chr1:3568669−3568722−Beta_values
A431
MCF7
Beta
1.00
0.75
0.50
0.25
0.00
3568660
3568670
3568680
3568690
3568700
3568710
3568720
position (bp)

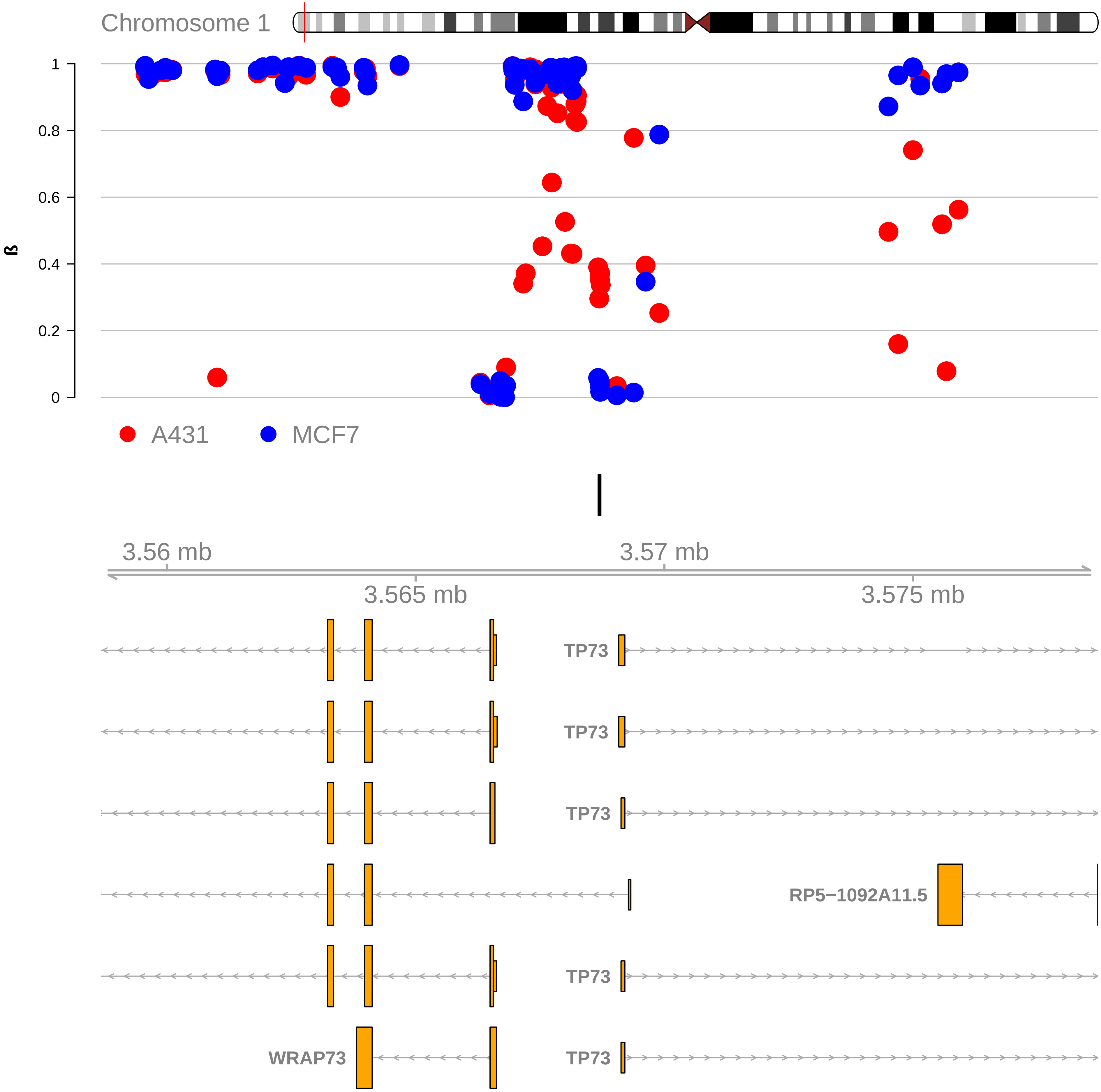

Chromosome 1
ß
1
0.8
0.6
0.4
0.2
0
A431
MCF7
3.56 mb
3.57 mb
3.565 mb
3.575 mb
TP73
TP73
TP73
RP5−1092A11.5
TP73
WRAP73
TP73
